# Comparative Genomic and Microenvironmental Profiles of Hereditary and Sporadic TNBC in Colombian Women

**DOI:** 10.3390/biology14121706

**Published:** 2025-11-30

**Authors:** Yina T. Zambrano-Ordoñez, Alejandro Mejía-Garcia, Julieta M. Ramírez-Mejía, Hsuan M. Tsao, Paula D. Morales-Suárez, Laura Rey-Vargas, Wendy J. Montero-Ovalle, Carlos A. Huertas-Caro, Patricia Lopez-Correa, Julián C. Riaño-Moreno, Juliana L. Rodriguez, Maria Carolina Sanabria-Salas, Luis G. Carvajal-Carmona, I. King Jordan, Silvia J. Serrano-Gomez, Liliana Lopez-Kleine, Carlos A. Orozco

**Affiliations:** 1 Grupo de Investigación Biología del Cáncer, Instituto Nacional de Cancerología, Bogotá 110111, Colombia; jramirez@cancer.gov.co (J.M.R.-M.); pmorales@cancer.gov.co (P.D.M.-S.); lrey@cancer.gov.co (L.R.-V.); wmontero@cancer.gov.co (W.J.M.-O.); chuertas@cancer.gov.co (C.A.H.-C.); macsanabriasa@unal.edu.co (M.C.S.-S.); silviajserrano@gmail.com (S.J.S.-G.); corozco@cancer.gov.co (C.A.O.); 2 Doctorado en Oncología, Departamento de Patología, Universidad Nacional de Colombia, Sede Bogotá, Bogotá 111321, Colombia; 3 Human Genetics Department, McGill University, Montreal, QC H3A 0G4, Canada; alejandro.mejiagarcia@mail.mcgill.ca (A.M.-G.); hsuan.tsao@mail.mcgill.ca (H.M.T.); 4 Maestría en Genética Humana, Universidad Nacional de Colombia, Sede Bogotá, Bogotá 111321, Colombia; 5 Departamento de Patología Oncología Molecular, Instituto Nacional de Cancerología, Bogotá 110111, Colombia; plopez@cancer.gov.co (P.L.-C.); jcriano@cancer.gov.co (J.C.R.-M.); 6 Facultad de Medicina, Universidad Cooperativa de Colombia, Villavicencio 500004, Colombia; 7 Grupo de Investigación Clínica y Epidemiológica, Instituto Nacional de Cancerología, Bogotá 110111, Colombia; jlrodriguez@cancer.gov.co; 8 Departamento de Ginecología y Obstetricia, Universidad Nacional de Colombia, Sede Bogotá, Bogotá 111321, Colombia; 9 Departamento de Ginecología y Obstetricia, Oncología Ginecológica–Fundación Santa Fe de Bogotá, Bogotá 110111, Colombia; 10 Department of Biochemistry and Molecular Medicine, University of California Davis, Davis, CA 95616, USA; lgcarvajal@ucdavis.edu; 11 Georgia Institute of Technology, Bioinformatics Department, Atlanta, GA 30332, USA; king.jordan@biology.gatech.edu; 12 Departamento de Estadística, Universidad Nacional de Colombia, Bogotá 111321, Colombia; llopezk@unal.edu.co; 13 Grupo de investigación en Oncología Traslacional, Instituto Nacional de Cancerología, Bogotá 110111, Colombia

**Keywords:** TNBC, hereditary breast cancer, gene expression profiling, Colombian/Latin American population

## Abstract

Breast cancer (BC) is a heterogeneous disease, and triple-negative breast cancer (TNBC) is the most aggressive and immunogenic subtype. A significant proportion of TNBC cases are linked to hereditary cancer syndromes involving pathogenic germline variants, most commonly in *BRCA1/2*. However, few studies have compared hereditary and sporadic TNBC in admixed populations. In this study, molecular and immunological features were analyzed through the analysis of 62 Colombian TNBC samples (20 hereditary and 42 sporadic cases) by RNA sequencing to identify molecular and immune differences. We used an external validation cohort of 16 TCGA TNBC cases (8 BRCA-mutated and 8 non-mutated) to replicate our findings. **Results:** We found a set of 921 differentially expressed genes (DEGs) between hereditary and sporadic TNBC. Hereditary tumors were enriched for pathways related to extracellular matrix (ECM) remodeling, structural components, and DNA damage response and exhibited a more immunologically active tumor microenvironment compared to sporadic tumors. LASSO logistic regression identified 23 genes with discriminatory potential, showing that hereditary tumors are characterized by complex immune regulation, inflammatory processes, and activation of key oncogenic pathways. **Conclusions:** Hereditary TNBC is characterized by molecular and biological functions linked to ECM remodeling and its constituents and an active immune microenvironment. This integrated molecular–immune profile provides insight into the distinct biology of hereditary tumors in admixed populations.

## Simple Summary

Triple-negative breast cancer (TNBC) is an aggressive subtype of breast cancer with limited therapeutic options. A proportion of cases are hereditary, mainly due to inherited variants, while most are sporadic and arise without a clear genetic cause. We compared hereditary and sporadic TNBC tumors from Colombian patients to explore their molecular and immune characteristics. Our study revealed that hereditary tumors display distinct biological features, including extracellular matrix remodeling and heightened immune activity, compared with sporadic tumors. These insights highlight the unique biology of hereditary tumors in diverse populations and may guide the design of more precise and personalized treatments in the future.

## 1. Background

Breast cancer (BC) is a heterogeneous disease that comprises four intrinsic molecular subtypes, Luminal A, Luminal B, Her2-enriched and triple-negative (TNBC) [1,2], each with distinct biological features and clinical behaviors [3,4,5]. TNBC, representing 15–20% of all breast cancers [6], is the most aggressive subtype of BC, characterized by the lack of estrogen receptor (ER), progesterone receptor (PR), and human epidermal growth factor receptor 2 (HER2) expression [7,8,9,10]. Although it can occur across all age groups, TNBC is more frequently diagnosed in younger women compared to other subtypes [11,12]. Clinically, it is associated with poor prognosis, high recurrence rates, and limited targeted therapeutic options [8,13,14].

A substantial proportion of TNBC cases, particularly among younger women, are associated with hereditary cancer predisposition syndromes [15], most commonly involving pathogenic or likely pathogenic (P/LP) germline variants in *BRCA1* or *BRCA2* genes. These variants are present in every cell from conception, can be transmitted to offspring, and confer a lifelong increased predisposition to cancer in carriers [16]. *BRCA1* variants occur in approximately 20–30% of TNBC cases and confer a lifetime risk of developing TNBC greater than 50% [17,18], whereas *BRCA2* variants are less frequent, occurring in about 5–10% of TNBC cases [19,20]. Additionally, variants in other homologous recombination repair (HRR) genes have also been implicated in a subset of cases [21,22]. Consistent with these findings, a report from the Colombian National Cancer Institute showed that TNBC cases had the highest diagnostic yield for a hereditary cancer syndrome (HCS), with 31.6% of patients carrying pathogenic variants [23]. *BRCA1* and *BRCA2* were the most frequently mutated genes, displaying a strong genotype–phenotype correlation with TNBC. Likewise, non-BRCA genes such as *PALB2* and *RAD51D* were also recurrently mutated in TNBC cases [24]. In contrast, sporadic cases develop in individuals without inherited cancer-predisposing variants, in whom oncogenic variants are acquired somatically in a single cell or cell lineage later in life. These variants accumulate over time, leading to a malignant phenotype only after sufficient genetic and epigenetic alterations have occurred [25]. Despite the clear etiological differences between hereditary and sporadic cases, few studies have systematically explored the downstream transcriptomic differences between these presentations.

On the other hand, it has been reported that TNBC is more immunogenic than other BC subtypes, characterized by higher levels of tumor-infiltrating lymphocytes (TILs) and increased expression of immune checkpoints, which influence both immunotherapy responsiveness and disease progression [26,27,28,29]. For instance, greater enrichment of plasma cells in the tumor microenvironment (TME) has been associated with improved prognosis, whereas higher levels of central memory CD4+ T cells (Tcm) have been linked to worse relapse-free survival, highlighting that not all immune infiltration is uniformly beneficial [30]. The immunogenic nature of TNBC may be modulated by its underlying germline variants in the tumor suppressor genes like *BRCA1* and *BRCA2*. Tumors associated with deleterious BRCA germline variants are more immunogenic compared to those associated with wild-type *BRCA1/2* [19,20], and beyond BRCA alterations, other germline variants in cancer predisposition genes may also shape the TME [31,32].

Although only a few molecular distinctions between hereditary and sporadic TNBC have been reported [33,34,35,36], most studies evaluating transcriptomic or molecular differences in BRCA-associated cancers have focused on ovarian cancer or combined cohorts of breast and ovarian tumors [37]. Furthermore, many BC studies do not distinguish between molecular subtypes of BC [24,38], despite growing evidence that each subtype exhibits distinct biological and immunological characteristics. A major challenge lies in the limited representation of admixed populations and the lack of studies integrating molecular and immune profiling to distinguish hereditary from sporadic TNBC. To address these gaps, this study investigated whether hereditary TNBC exhibits distinct molecular and immune features compared to sporadic cases in Colombian women. To this end, we performed a comparative transcriptomic analysis and immune deconvolution. This integrative approach provides novel insights into the biological underpinnings of TNBC heterogeneity, particularly into molecular pathways and differences in TME between hereditary TNBC (H-TNBC) and sporadic TNBC (S-TNBC) in admixed populations.

## 2. Materials and Methods

### 2.1. Study Samples

This study included 72 women diagnosed with TNBC who were enrolled in the Hereditary Cancer Program at the Colombian National Cancer Institute (NCI-Col) between 2018 and 2023 [23,39]. As part of the program, all patients received genetic counseling and underwent germline testing in accordance with the National Comprehensive Cancer Network (NCCN) guidelines (2018–2023) [23,39,40]. All patients provided written informed consent, and both clinical data and biological samples were collected. Ethical approval for the study was obtained from the Ethics Committee of the NCI-Col, ensuring full compliance with ethical standards and patient privacy protections. For this study, patients were classified into two groups based on the presence of P/LP germline variants in genes associated with hereditary breast cancer syndromes. The hereditary group (H-TNBC, N = 20) included patients carrying germline variants in breast cancer risk genes (*BRCA1*, *BRCA2*, *BARD1*, *CHEK2*, *PALB2*), as well as in *MUTYH* and *PMS2*, which have been reported in breast cancer cases and are involved in other DNA repair pathways (Base Excision Repair (BER), and Mismatch Repair (MMR)), although their association with breast cancer risk remains uncertain. While the sporadic group (S-TNBC, N = 42) comprised those without any pathogenic germline variants. Patients carrying P/LP variants in genes not directly associated with cancer risk or hereditary breast cancer were excluded from the study to minimize potential confounding effects of these variants on the results.

### 2.2. Ethical Considerations

This study was conducted in accordance with the Declaration of Helsinki and approved by the Ethics Committee of the Instituto National Cancer Institute, Bogotá, Colombia (C19010300455, approval date: 23 June 2020). All patients provided written informed consent, and both clinical data and biological samples were collected. Ethical approval for the study was obtained from the Ethics Committee of the National Cancer Institute of Colombia, ensuring full compliance with ethical standards and patient privacy protections.

### 2.3. Study Power and Sample Size

The sample size was estimated using a classical approach for comparing proportions, based on a two-tailed hypothesis test commonly applied in epidemiological and clinical studies. We assumed a baseline prevalence of germline mutations between 24% and 34%, as reported in previous studies, and an expected difference of approximately 5% between hereditary and sporadic TNBC cases, with a significance level (α) of 0.05 and statistical power (1 − β) of 80%. The final analyzed cohort comprised 62 patients (20 hereditary and 42 sporadic). The study remains an exploratory analysis: a first integrative effort to characterize the molecular and immunological landscape of hereditary and sporadic TNBC in an admixed Latin American population. Despite the limited sample size, the approach allows the identification of biologically meaningful differences and provides a foundational framework for future validation in larger cohorts.

### 2.4. Sample Collection and Sequencing

Blood samples were collected to determine germline variants using the TruSight™ Hereditary Cancer Panel (customized probe panel reference #20011891; Illumina Inc., San Diego, CA, USA) (Supplementary Table S1), with the MiSeq Sequencing System (Illumina Inc., San Diego, CA, USA) [41]. Details about germline DNA extraction, library preparation, and sequencing assays have been previously described [23,41]. RNA was extracted from formalin-fixed, paraffin-embedded (FFPE) tumor tissue blocks before any treatment, using the RecoverAll^TM^ Total Nucleic Acid Isolation extraction kit (Thermo Fisher Scientific, Waltham, MA, USA), following the manufacturer’s protocol. RNA was quantified with the Qubit dsDNA BR kit (Thermo Fisher Scientific, Waltham, MA, USA) and the NanoDrop™ 2000 equipment(Thermo Fisher Scientific, Wilmington, DE, USA), and the integrity and concentration were assessed using the Agilent 2100 Bioanalyzer with the High Sensitivity DNA Assay (Agilent Technologies, Santa Clara, CA, USA). Library preparation and sequencing were performed as a technical service at the University of California, Davis (UC-Davis) Genome Center (CA, USA). Libraries were prepared using the SMART-Seq Total RNA Pico Input with UMIs (ZapR Mammalian), according to the manufacturer’s recommendations, and final sequencing was carried out on the NovaSeqX 25B lane as PE150 (Illumina Inc, San Diego, CA, USA), generating paired-end reads with a depth of 50X to ensure sufficient coverage for transcript quantification.

### 2.5. Bioinformatic Processing and Quality Control of RNA-Seq Data

**Figure 1:**
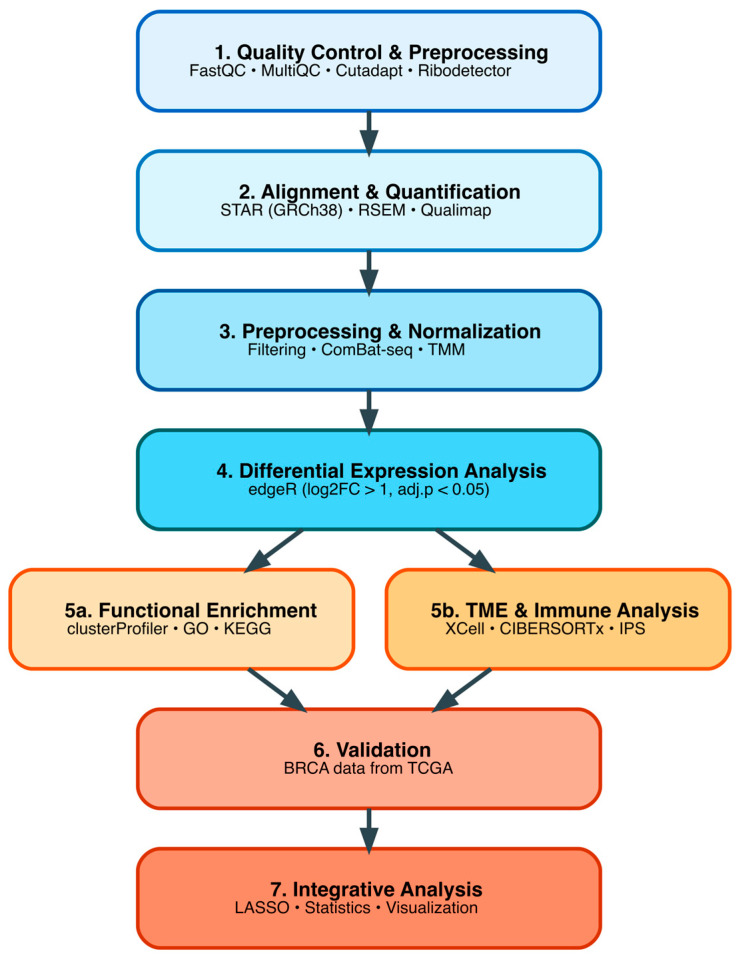
Bioinformatic and analytical workflow.

A total of 62 of the 72 sequenced samples passed all quality control (QC) due to RNA degradation and fragmentation. An overview of the bioinformatic pipeline applied in this study is shown in Figure 1. Prior to alignment, raw reads were analyzed using FastQC (v0.12.1) (https://www.bioinformatics.babraham.ac.uk/projects/fastqc/, accessed on 18 November 2025) and summarized using MultiQC (v1.24.1) (https://seqera.io/multiqc/, accessed on 18 November 2025). Adapter sequences were trimmed using Cutadapt (v4.9) (https://cutadapt.readthedocs.io/en/stable/, accessed on 18 November 2025) and Ribodetector (v3.1) (https://helmholtz.software/software/ribodetector, accessed on 18 November 2025). Read alignment of reads to the human reference genome GRCh38 was performed using STAR (v2.5.2) (https://research.stowers.org/cws/CompGenomics/Projects/star.html, accessed on 18 November 2025), using the Gencode v38 annotation file (gencode.v38.annotation.gtf). The alignment process focused on uniquely mapped reads, and only these were considered for downstream analysis to ensure the accuracy of gene expression quantification. Gene expression quantification was carried out using RSEM (RNA-Seq by Expectation-Maximization, v2.5.2) (https://github.com/deweylab/RSEM, accessed on 18 November 2025), which generated a raw count matrix for subsequent differential expression analysis. All bioinformatic analyses were performed on a high-performance computing server at Dr. King Jordan’s Laboratory, Georgia Institute of Technology (Atlanta, GA, USA), under a Linux environment with custom Bash commands for workflow automation and reproducibility.

### 2.6. Differential Gene Expression Analysis in Colombian Cohort

The raw count matrix obtained from RSEM was processed in RStudio (v2023.12.1.402) using Bioconductor to perform differential gene expression analysis by three preprocessing steps. (1) Initially, to control for batch effects across experimental groups, ComBat-seq (from the *sva* package) was applied to the raw count matrix prior to normalization and differential expression analysis. (2) We performed sample quality control to identify and exclude outliers. Pairwise Pearson correlation coefficients were calculated within each group, and only samples with a mean intra-group correlation ≥ 0.6 were retained, a threshold used to ensure sample concordance in RNA-seq datasets. (3) We performed gene filtering. Genes with extremely high counts (>1 million reads) and those with zero counts across all samples were removed. Genes with very low expression were filtered out by retaining only those with expression values ≥ 10 counts in at least two samples. After these steps, 62 samples (H-TNBC = 20, S-TNBC = 42) and 17,061 genes were retained for downstream analyses. Gene expression data were normalized using the TMM (Trimmed Mean of M-values) method to account for differences in sequencing depth and library composition. Log2-transformed counts per million (CPM), adjusted by the TMM normalization factors, were then used for downstream analyses. Differential gene expression analysis was performed using the *edgeR* package. Genes were considered significantly differentially expressed if they had log2FC > 1 and an adjusted *p*-value < 0.05.

### 2.7. Functional Enrichment Analysis

Functional annotation of differentially expressed genes (DEGs) was performed using Gene Ontology (GO) and Kyoto Encyclopedia of Genes and Genomes (KEGG) enrichment analyses. DEGs were stratified into upregulated and downregulated gene sets and analyzed independently to identify overrepresented biological processes and signaling pathways. All enrichment analyses were conducted in R using the *clusterProfiler*, *org.Hs.eg.db* and *enrichplot* packages. The results were visualized using dot plots to summarize key biological functions associated with the transcriptional changes observed in H-TNBC and S-TNBC tumors. To ensure methodological transparency and reproducibility, all enrichment analyses were performed using open-source R packages.

### 2.8. Tumor Microenvironment Deconvolution Analysis in Colombian Cohort

The prepared gene expression data (62 samples and 17,061 genes) was further normalized to TPM (Transcripts Per Million) values independently from the differential expression analysis, as required by immune deconvolution algorithms. TPM normalization ensures comparability of expression levels across samples and was used as input for downstream TME inference analyses. To infer the immune composition of tumor samples, we employed computational deconvolution methods XCell [42] and CIBERSORTx [43] to characterize immune cell infiltration across samples. These approaches have been benchmarked in large-scale assessments, demonstrating their reliability in estimating cellular fractions and immune signaling activity from bulk RNA-seq data [44]. Additionally, we computed the Immunophenoscore (IPS) as described by Charoentong et al. Briefly, gene expression values were normalized and converted into Z-scores, which were then aggregated into immune checkpoints (ICPs) using the original weights defined by [45]. The arithmetic sum of weighted category scores yielded an overall IPS, where higher values indicate greater predicted responsiveness to ICP blockade. For categorical comparisons, IPS was dichotomized into high (≥5) and low (<5), consistent with the thresholds defined in The Cancer Immunome Atlas (TCIA) framework. XCell and CIBERSORTx were selected because they provide finer immune and stromal resolution, are compatible with smaller non-TCGA datasets, and allow user-defined references—features particularly suited for our Colombian cohort.

### 2.9. DEG Validation Analysis Using TCGA TNBC Cohort

To validate our transcriptomic and TME findings, we leveraged publicly available datasets from The Cancer Genome Atlas (TCGA). Specifically, we used data included in a large-scale pan-cancer study (PMID: 32396860) [46]. Samples were classified as H-TNBC if they harbored P/LP germline variants in *BRCA1/2*, as reported in the TCGA metadata. S-TNBC cases were defined as those without germline variants in BC susceptibility genes according to TCGA metadata. Based on these criteria, we selected 16 TNBC tumor samples, including 8 H-TNBC and 8 S-TNBC. Unlike our Colombian cohort, in which hereditary cases were defined based on a broader set of breast cancer-associated genes, the TCGA hereditary group was restricted to carriers of germline variants in BRCA1/2 only. Raw counts from the 16 cases were normalized and processed following the same bioinformatic pipeline applied to the Colombian cohort. To validate these findings, IPS values were obtained from the Cancer Immunome Atlas (TCIA) for matched TNBC patients using TCGA barcodes. IPS values were similarly classified into high (≥5) and low (<5), following the official TCIA categorization. Subsequently, we performed a comparative analysis of DEGs between both cohorts. Shared DEGs were identified. To evaluate the consistency of DEGs between our cohort and TCGA data, we computed the Spearman correlation coefficient (ρ) between the Log2FC of the overlapping DEGs. This non-parametric approach was chosen due to its robustness to outliers and non-normal distributions, providing a reliable measure of rank-based concordance between both datasets.

Detailed bioinformatics outputs, including RNA-seq quality control metrics, variant characterization, functional enrichment results, and LASSO gene selection, are available in Supplementary Figures S1–S3 and Supplementary Tables S2 and S3.

### 2.10. Statistical Analysis

Comparisons between categorical clinical variables across groups (H-TNBC vs. S-TNBC) were performed using Fisher’s exact test or Chi-squared test, depending on sample size and distribution assumptions. Group comparisons for immune deconvolution outputs were assessed using the two-sided Wilcoxon rank-sum test. Multiple testing correction was applied using the Benjamini–Hochberg method where appropriate. Additionally, we performed least absolute shrinkage and selection operator (LASSO) logistic regression, a penalized regression method that performs both variable selection and regularization by constraining the sum of the absolute values of the model coefficients. This approach reduces overfitting and identifies the most informative predictors to discriminate between H-TNBC and S-TNBC. The analysis was implemented using the intersection of DEGs shared between the Colombian and TCGA cohorts to evaluate their association with tumor condition (H-TNBC vs. S-TNBC). The goal was to identify a minimal gene signature capable of discriminating H-TNBC from S-TNBC tumors. The model was fitted using a 10-fold cross-validated LASSO approach (*cv.glmnet*) (https://cran.r-project.org/web/packages/glmnet/index.html, accessed on 18 November 2025), with a binomial outcome (0 = S-TNBC, 1 = H-TNBC). Gene selection was based on non-zero coefficients obtained at the optimal penalty parameter (λ), determined by minimizing the cross-validation error (*lambda.min*). This approach ensures that the selected features reflect robust and reproducible biological signals across cohorts. All statistical analyses and visualization graphics were conducted in RStudio (v2023.12.1.402) (https://posit.co/download/rstudio-desktop/, accessed on 18 November 2025).

## 3. Results

### 3.1. Clinical and Pathological Characteristics of Colombian Cohort

We analyzed a sample of 62 Colombian women diagnosed with TNBC at NCI-Col. Patients were stratified into groups based on germline testing results: the H-TNBC group (N = 20), which included women harboring P/LP variants in BC susceptibility genes (Supplementary Table S1), and the S-TNBC group (N = 42), composed of patients without any P/PL variants. To explore whether these groups differed in their clinical, pathological, and sociodemographic characteristics, we conducted comparative statistical analyses between H-TNBC and S-TNBC. Overall, no statistically significant differences were found across most variables (Table 1). The only statistically significant difference was observed in lymph node involvement, where 86% of sporadic cases had lymph node metastasis compared to 55% in the hereditary group (*p* = 0.028) (Table 1).

This suggests more aggressive regional involvement at diagnosis among S-TNBC in this cohort. At first consultation, all distant metastases in the sporadic group corresponded to regional lymph nodes, whereas in the hereditary group, lung and pleural involvement was observed, though these trends did not reach statistical significance.

In the H-TNBC group, BRCA1 was the most frequently affected gene, with P/LP variants identified in 10 patients. The well-known recurrent variants previously reported in Colombian [23] BRCA1 variants c.5123C>A p. (Ala1708Glu) and c.1674delA (p. Gly559ValfsTer) were observed in three and two unrelated patients, respectively, while the other variants were detected only once (Supplementary Figure S2).

### 3.2. Bioinformatic Data Preprocessing and Quality Control of Sequences

RNA-seq QC confirmed overall high sequencing and mapping performance across the 62 retained samples. As shown in Supplementary Figure S1, most reads were uniquely mapped to the human reference genome (GRCh38), with low duplication rates and consistent coverage across exonic regions. Minor variability in mapping efficiency was observed but remained within acceptable thresholds for downstream differential expression analysis. These results validate the reliability of the processed dataset for subsequent transcriptomic and functional analyses.

### 3.3. Differential Gene Expression Analysis in Colombian Women Cohort

**Figure 2:**
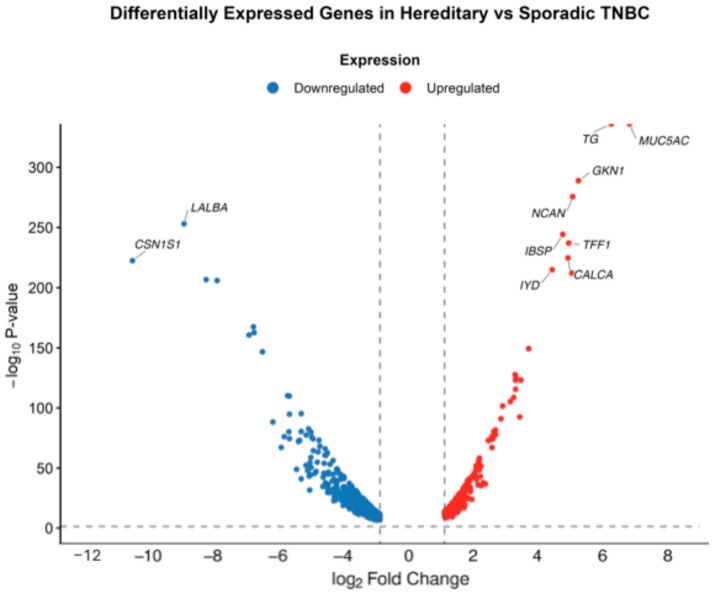
Volcano plot of differentially expressed genes between hereditary and sporadic TNBC cases in the Colombian cohort. Volcano plot displaying 921 DEGs between H-TNBC and S-TNBC. Each point represents a gene. Red dots represent significantly upregulated DEGs, and blue dots indicate significantly downregulated genes. The top significant DEGs are labeled.

Global transcriptional profiling revealed distinct gene expression patterns between hereditary and sporadic Colombian patients with TNBC. After filtering and normalization of raw RSEM counts, differential gene expression analysis identified a total of 921 DEGs (Log2FC > 1; *p* adjusted value < 0.05). Among these, 249 genes were upregulated, and 672 genes were downregulated in H-TNBC compared to S-TNBC. Expression profiles of these genes are shown in the volcano plot (Figure 2). Among the top DEGs, we identified the downregulation of LALBA, CSN1S1 and KRT13, and the upregulation of *TG*, *MUC5AC*, *GKN1/2*, *NCAN*, *IBSP*, *TFF1*, *CALCA* and *IYD*. Several of these genes have been previously linked to tissue-specific functions and tumor biology, highlighting their potential contribution to the molecular differences observed between hereditary and sporadic TNBC.

### 3.4. Functional Enrichment Analysis in Colombian Women Cohort

**Figure 3:**
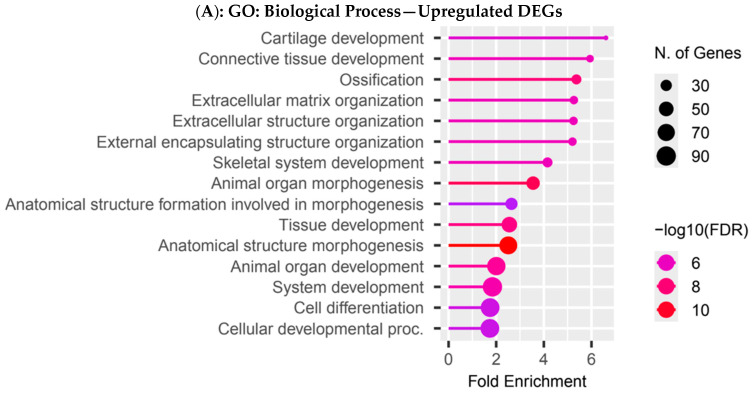

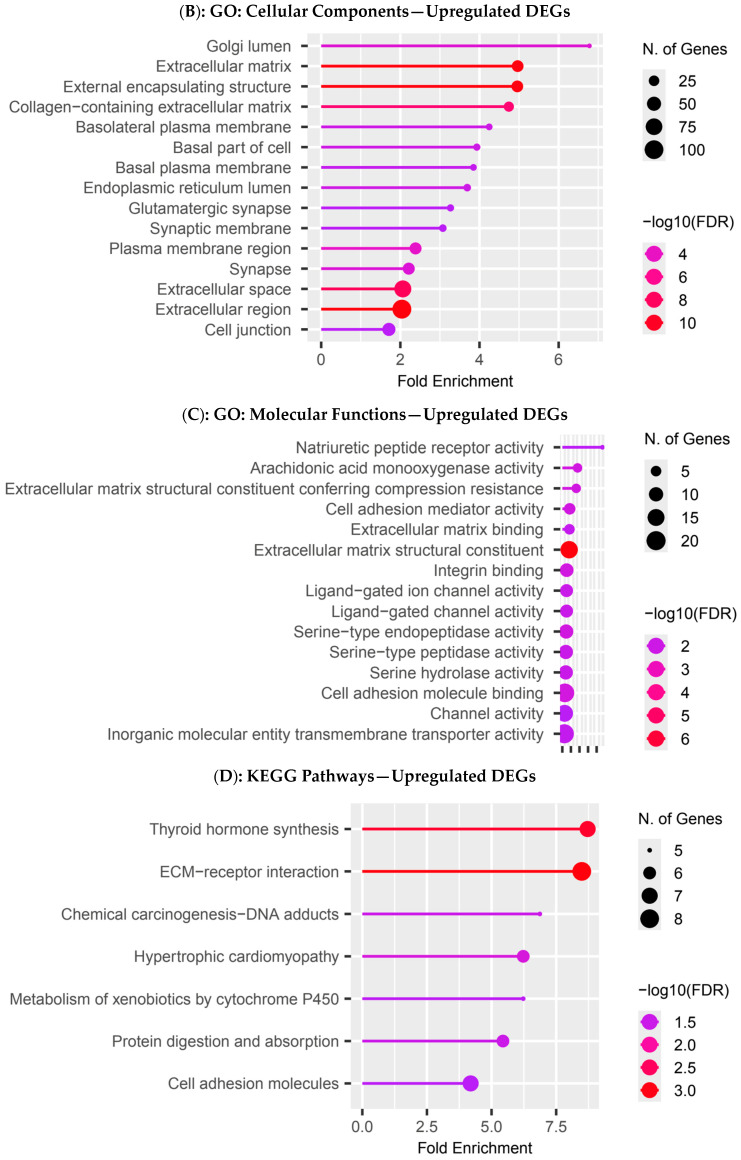
Functional enrichment analyses in the Colombian cohort. Functional enrichment compared H-TNBC vs. S-TNBC. (**A**) GO biological process. (**B**) GO cellular component. (**C**) Molecular functions. (**D**) KEGG pathways. Each dot represents an enriched category, with size indicating the number of genes and color representing statistical significance.

To better understand the biological differences observed between hereditary and sporadic tumors in the Colombian cohort, we performed functional enrichment analysis using upregulated and downregulated DEGs separately (Figure 3A–D). Upregulated DEGs in H-TNBC were significantly enriched in biological processes (BP) related to extracellular matrix (ECM) remodeling, including connective tissue and other related processes, suggesting increased stromal activity (Figure 3A). For the cellular component (CC), upregulated DEGs were enriched in components associated with the extracellular matrix, collagen-containing structures, and plasma membrane structures (Figure 3B). For the molecular functions (MF), upregulated genes displayed enrichment in extracellular matrix structural constituents, integrin binding, and ligand-gated ion channel activity, underscoring roles in cell–matrix adhesion and signaling (Figure 3C). KEGG pathway enrichment showed upregulation of pathways involved in ECM–receptor interaction, thyroid hormone synthesis, and chemical carcinogenesis–DNA adducts, emphasizing processes involved in extracellular matrix remodeling and potential endocrine signaling and genotoxic stress responses in H-TNBC (Figure 3D). Conversely, downregulated DEGs (Supplementary Figure S3) were enriched in BPs related to skin and epidermal development, keratinocyte differentiation, and epithelial cell differentiation (Supplementary Figure S3A). In the CC category, these genes localized to synaptic and postsynaptic membranes, GABA-A receptor complexes, and ion channel clusters (Supplementary Figure S3B). MF terms included ion channel activity and neurotransmitter receptor activity (Supplementary Figure S3C). KEGG pathway enrichment showed association with signaling and metabolic pathways, GABAergic and serotonergic synapses, and neuroactive ligand–receptor interaction (Supplementary Figure S3D).

### 3.5. Differential Immune Cell Infiltration in Colombian Patients

**Figure 4:**
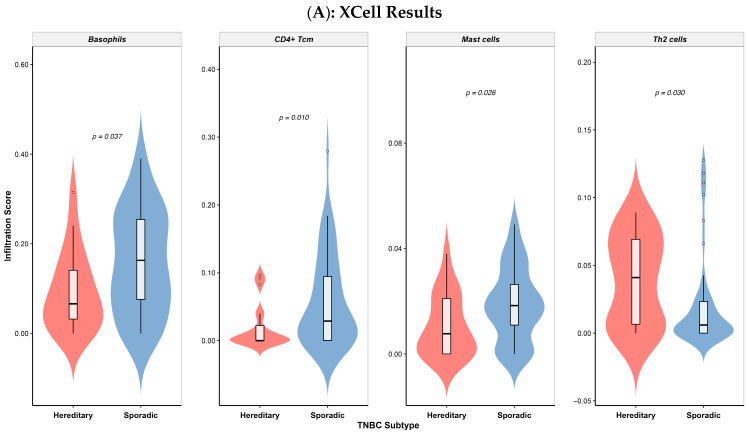

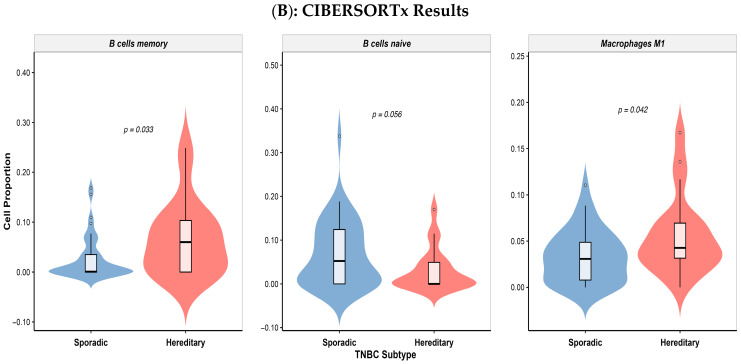
TME differences between hereditary and sporadic TNBC Colombian patients. Estimated proportions of immune cell types in H-TNBC and S-TNBC. (**A**) XCell analysis. (**B**) CIBERSORT analysis. Comparisons between groups were assessed using the Wilcoxon rank-sum test; *p*  <  0.05 was considered statistically significant.

To explore differences in immune cell infiltration between hereditary and sporadic TNBC tumors, we applied two complementary deconvolution algorithms XCell and CIBERSORTx to our batch-corrected and TPM-normalized expression data. The goal was to capture distinct features of the immune microenvironment associated with the presence or absence of P/LP germline variants in BC risk genes (*BRCA1*, *BRCA2*, *BARD1*, *CHEK2*, *MUTYH*, *PALB2*, and *PMS2*). The XCell analysis revealed immune populations with significantly different distributions across groups (Figure 4A). Tumors from the S-TNBC group showed higher relative abundance of basophils (*p* = 0.037), central memory CD4^+^ T cells (Tcm) (*p* = 0.0098), and mast cells (*p* = 0.026). In contrast, Th2 cells were more enriched in H-TNBC samples (*p* = 0.029), potentially reflecting an immune polarization skewed toward humoral or anti-inflammatory responses. Complementary findings emerged from the CIBERSORTx analysis (Figure 4B), which highlighted greater infiltration of memory B cells (*p* = 0.033) and pro-inflammatory M1 macrophages (*p* = 0.042) in H-TNBC tumors. Overall, H-TNBC tumors seem to display a more mature and activated immune microenvironment, while sporadic cases are marked by a less differentiated, potentially tumor-promoting immune profile. On the other hand, no significant differences in IPS were observed between H-TNBC and S-TNBC in the Colombian cohort (Supplementary Table S2).

### 3.6. Validation Using TCGA Breast Cancer Cohort

**Figure 5:**
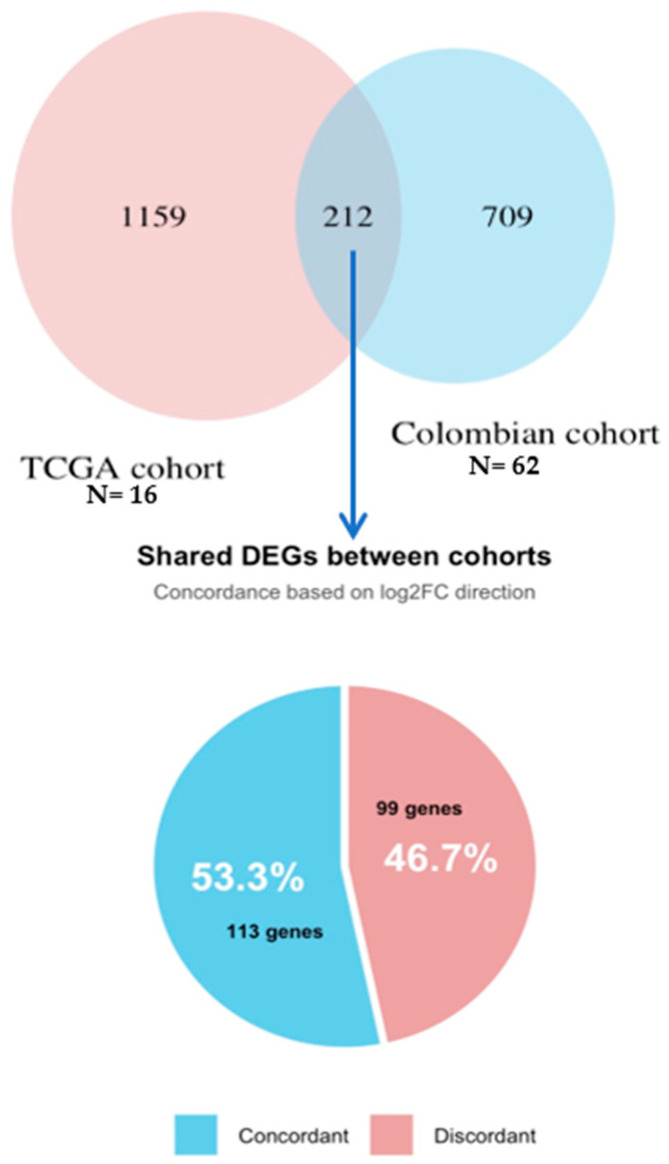
Overlap and concordance of differentially expressed genes between the Colombian and TCGA cohorts. The Venn diagram (**top**) shows the overlap of DEGs identified in the Colombian cohort and the TCGA cohort. The pie chart (**bottom**) represents the concordance in the direction of expression changes (Log2FC) among these shared DEGs: 113 genes showed concordant regulation (either upregulated or downregulated in both cohorts).

**Figure 6:**
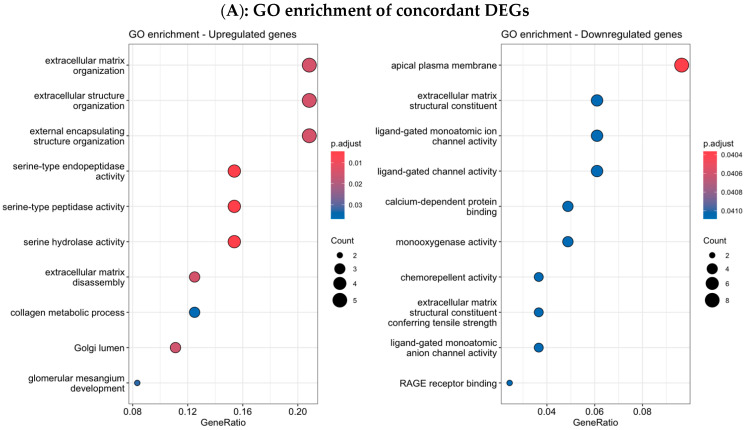

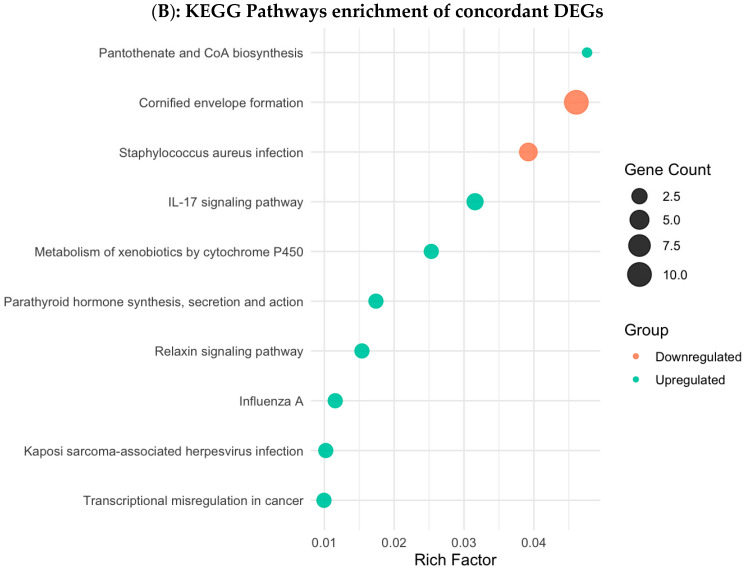
Functional enrichment analysis and KEGG pathways in 113 shared DEGs between cohorts. (**A**) GO enrichment analysis. (**B**) KEGG pathways enriched. Each dot represents an enriched category, with size indicating the number of genes and color representing statistical significance.

Comparison of gene expression findings by comparing tumor DEGs from the Colombian cohort with DEGs from the TCGA cohort revealed 113 concordant DEGs in both datasets (Figure 5) of which 27 were upregulated and 86 downregulated. The Log2FC correlation showed a positive correlation between our differential expression results and those from TCGA for the concordant genes (Spearman’s rho = 0.59, *p* < 2.2 × 10^−16^). Genes upregulated in our cohort tended to be upregulated in TCGA as well, supporting the robustness and reproducibility of our findings. To better understand the biological relevance of the concordant gene set between cohorts, we performed functional enrichment analysis (Figure 6). GO analysis (Figure 6A) showed that upregulated genes were predominantly involved in ECM organization, collagen metabolic processes, and other functions linked to tumor invasion, metastasis and ECM remodeling. On the other hand, downregulated DEGs were enriched in synaptic signaling, ion channel activity and receptor binding. Finally, KEGG pathway analysis (Figure 6B) identified several cancer-related pathways, including IL-17 signaling, transcriptional cancer-related pathways and metabolism of xenobiotics by CYP450 (cytochrome P450). For IPS, similarly, in the Colombian cohort, the TCGA revealed no significant distinction between H-TNBC and S-TNBC (Supplementary Table S2). These findings suggest that, in both datasets, inherited and sporadic TNBC may display comparable levels of immunogenicity and predicted response to checkpoint inhibition as measured by IPS.

**Figure 7:**
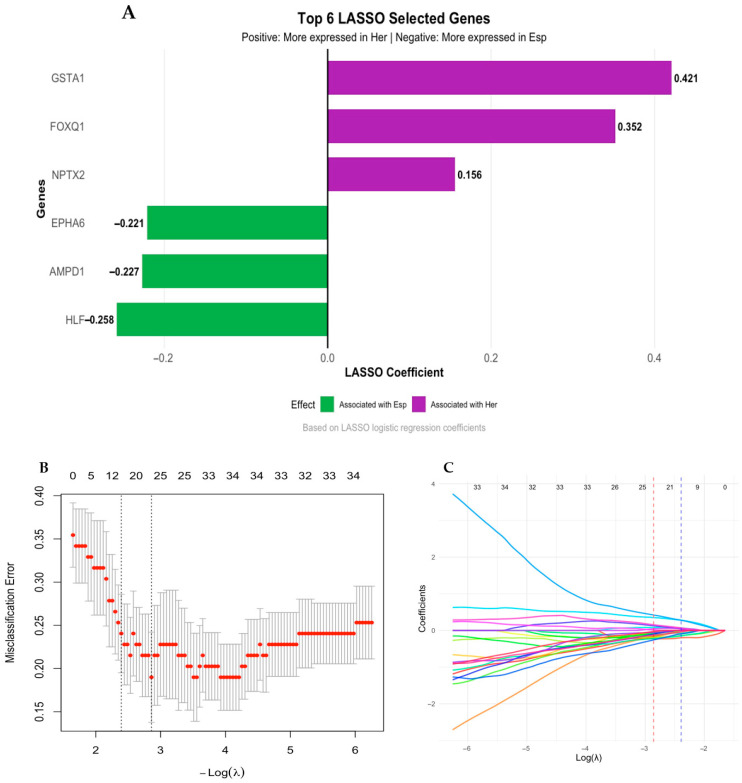
LASSO logistic regression distinguishing hereditary from sporadic TNBC. (**A**) Bar plot displaying the top six gene coefficients selected by LASSO logistic regression. Positive values represent genes with higher expression in H-TNBC (purple); negative values indicate genes more expressed in S-TNBC (green). (**B**) Cross-validation plot of the LASSO logistic regression showing the misclassification error rate as a function of the penalty parameter λ. The dotted lines indicate the value of λ with the minimum misclassification error and the value of λ within one standard error of the minimum. The numbers at the top indicate the number of variables selected at each point. (**C**) Coefficient trajectories as a function of log(λ). Each line represents a predictor, and non-zero values at the optimal λ correspond to the variables selected to discriminate between hereditary and sporadic TNBC.

Furthermore, we applied a LASSO logistic regression model. This approach selected 23 genes with discriminative potential between H-TNBC and S-TNBC tumors (Supplementary Table S3), among which six were retained with non-zero coefficients at the optimal λ (Figure 7B,C). Notably, genes such as *GSTA1*, *FOXQ1* and *NPTX2* presented positive coefficients, suggesting higher expression in H-TNBC (Figure 7A), while *HLF*, *AMPD1* and *EPHA6* exhibited negative coefficients (Figure 7A), demonstrating reduced expression in H-TNBC compared to S-TNBC. These discriminatory patterns were consistent across stability paths visualized in the coefficient trace plot (Figure 7C), supporting the biological relevance of these markers. These genes identified by LASSO are involved in key biological processes and pathways (Table 2). GO terms in the BP category highlighted roles in immune regulation, including regulation of IL-17 production, myeloid leukocyte cytokine production, and negative regulation of response to cytokine stimulus. Additionally, genes were associated with cellular mechanisms such as cell–cell adhesion, regulation of protein secretion, and cell activation involved in the immune response. In the MF category, enriched terms included cytokine receptor binding, receptor ligand activity, transferase activity, and kinase activity. Finally, KEGG pathway analysis further supported involvement in immune-related and oncogenic signaling, including the cytokine–cytokine receptor interaction, TNF signaling pathway, NF-kappa B signaling pathway, apoptosis, and broader pathways in cancer (Table 2).

## 4. Discussion

There is growing interest in characterizing the molecular differences between hereditary and sporadic tumors. Most studies focus on understanding differences in transcriptome profiles of ovarian cancer patients bearing variants in *BRCA1* or *BRCA2* [33,37]. In this study, we aimed to investigate the molecular and immune differences between H-TNBC and S-TNBC in Colombian women using a reproducible bioinformatic framework that ensured the robustness and reliability four the analysis. We found a set of 921 DEGs between H-TNBC and S-TNBC in the Colombian cohort. In contrast, Hedenfalk et al., who analyzed a smaller cohort of seven sporadic, seven *BRCA1* and eight *BRCA2* breast cancers, reported a set of 51 DEGs [33]. The broader definition of hereditary tumors in our study, which included carriers of P/LP variants in breast cancer risk genes beyond the *BRCA1/2* genes, may partly account for the larger number of DEGs observed. These findings not only reinforce the existence of transcriptomic distinctions between hereditary and sporadic breast cancers but also expand the perspective of hereditary TNBC beyond the BRCA genes traditionally recognized as central to disease risk. As for the functional annotation, ECM remodeling and signaling pathway activation were enriched in H-TNBC compared to S-TNBC, and these patterns were consistently observed in both the Colombian and TCGA cohorts. This is consistent with the existing evidence supporting the role of ECM as a dynamic microenvironment that regulates key cancer processes [47,48]. Furthermore, in our cohort, upregulated genes in H-TNBC were enriched for molecular functions such as ECM constituents with integrin binding, and ligand-gated ion channel activity, which is consistent with results showing that integrin interactions are key mediators of cellular adhesion and migration in cancer [49,50], which could be related to hereditary genetic alterations that modulate the expression of cell adhesion and ECM genes, contributing to a distinct molecular profile compared to sporadic ones.

KEGG pathway enrichment analysis in the Colombian cohort revealed enrichment in thyroid hormone synthesis and chemical carcinogenesis–DNA adduct pathways. While these processes are not highlighted in TNBC biology, emerging evidence supports their potential relevance in tumor progression and therapeutic response. For example, it has been reported that alterations in endocrine-related tumor suppression mechanisms specific to hereditary tumors modulate proliferative signaling, induce apoptosis, reduce aggressive phenotypes, and can improve the response to chemotherapy [51,52,53,54]. Similarly, the chemical carcinogenesis–DNA adduct pathway involves the accumulation of DNA adducts, which has been associated with genomic instability, increased mutagenesis, and sensitivity to DNA-damaging agents [55,56]. This is particularly relevant in the context of our cohort, which includes patients harboring germline variants in DNA repair genes. *BRCA1*, *BRCA2*, *PALB2*, *RAD51C*, and *CHEK2* are key components of the homologous recombination repair (HRR) pathway, while others such as *MUTYH* are implicated in base excision repair. The inefficient DNA repair resulting from variants in critical genes may make hereditary tumors more susceptible to DNA adduct accumulation and the ensuing genomic instability [57]. Indeed, TNBCs, especially those with germline alterations in genes like *BRCA1/2* and other HRR genes, are well recognized to harbor DNA repair defects that drive genomic instability and sensitize tumors to DNA-damaging agents such as PARP inhibitors and platinum-based chemotherapy [57,58]. Interestingly, in the analysis of both cohorts, we detected a downregulation of neurotransmitter-related pathways, including GABAergic and serotonergic synapse signaling. While we did not collect longitudinal clinical data on brain metastases in our cohort, this finding aligns with evidence suggesting that TNBC cells and brain metastases can retain neural signaling pathways. The reduced expression of genes involved in these neurotransmission systems may alter TME interactions, with potential implications for tumor progression, metastasis, and treatment response [59,60,61].

Regarding the TME analysis, we found evidence that H-TNBCs are enriched in Th2 cells, memory B cells, and M1 macrophages. Considering the study of Jacenik et al., 2023, the elevated presence of Th2 cells in H-TNBC could promote the activation and recruitment of M1 macrophages, contributing to a more effective anti-TME [62]. Additionally, tumors carrying germline variants in DNA repair genes have higher mutational burden (TMB) [63]. Increased TMB has been associated with the infiltration of effector immune cells, including M1 macrophages, and they also correlate with the infiltration of regulatory T cells (Tregs) [64,65,66]. This is because an excessive inflammatory response requires a subsequent regulation mechanism. For example, the intense initial inflammatory activity of M1 macrophages in tumors is regulated by Th2 cells and cytokines such as IL-4 and IL-10 to achieve an effective yet controlled anti-tumor response. This dynamic regulation prevents uncontrolled inflammation and protects the tissue microenvironment [67,68].

Likewise, the high presence of memory B cells in the TME of H-TNBC cases may reflect the heightened immunogenicity driven by genomic instability and increased neoantigen load, particularly in cases harboring germline defects in DNA repair genes such as *BRCA1/2* [69,70]. However, the presence and functional profile of B cells vary according to the molecular and genetic context of the tumor [71]. Within the TME, memory B cells can contribute to immunosurveillance through the production of tumor-specific antibodies and by orchestrating both cellular and humoral immune responses [66,72]. Therefore, our results are aligned with the scientific evidence which associates inherited variants with a more immunogenic TME and potentially better response to immunotherapies. In contrast, sporadic tumors usually present a less differentiated and sometimes pro-tumoral immune profile [66,73]. However, when assessing the IPS and evaluating ICP, we observed no significant difference between H-TNBC and S-TNBC. This finding suggests that despite differences in immune cell composition and molecular differences, the overall predicted responsiveness to ICP inhibitors may be comparable between these groups. While H-TNBC cases are frequently enriched in P/LP variants, which have been associated with higher tumor mutational burden and increased neoantigen load [63], our findings indicate that these genomic features may not translate into marked differences in IPS when compared with sporadic TNBC. Consistent with previous reports, IPS appears to reflect the overall immunogenicity of TNBC rather than its hereditary background [74,75].

Finally, a 23-gene set with discriminatory potential between H-TNBC and S-TNBC selected by LASSO is in line with current scientific evidence showing that hereditary tumors are characterized by complex immune regulation, inflammatory processes, and activation of key oncogenic pathways [67,76,77]. Together, these findings support the critical role of the interaction between immune and oncogenic processes in the H-TNBC phenotype, offering potential targets for targeted and immunomodulatory therapies.

Beyond the biological findings, it is important to acknowledge that our analysis was conducted in an admixed Colombian population. While this may limit the direct extrapolation of the results to other groups, it also represents a crucial step toward addressing the persistent underrepresentation of Latin American populations in cancer genomics. The predominance of European ancestry within our cohort offers partial comparability with international datasets, yet the unique admixture background provides a distinctive lens through which to interpret TNBC biology in a real-world, non-European context. Expanding similar integrative studies across diverse ancestries will be essential to ensure equitable translational relevance of molecular oncology research. Looking forward, future studies should extend these findings through experimental validation of key molecular pathways and immune signatures, as well as through longitudinal analyses integrating clinical outcomes. Incorporating single-cell and spatial transcriptomic approaches, along with functional assays in cell lines or patient-derived models, will help unravel the mechanistic links between germline variants, immune remodeling, and TNBC progression.

## 5. Conclusions

This study reveals that H-TNBC harbor distinct molecular and immunological signatures compared to S-TNBC. Tumors from carriers of pathogenic or likely pathogenic germline variants were enriched for pathways related to extracellular matrix remodeling and neurotransmission signaling, potentially facilitating metastatic dissemination. Concomitantly, the tumor microenvironment displayed an active anti-tumor immune profile, with increased Th2 cells, M1 macrophages, and memory B cells, possibly reflecting enhanced neoantigen presentation driven by germline variants. These findings suggest that hereditary tumors engage in a coordinated structural and immune remodeling that may shape tumor evolution and therapeutic response. Validation in the TCGA cohort, although limited to BRCA1/2 status, supports that the hereditary component influences both the molecular architecture and immune dynamics of TNBC.

### 5.1. Future Perspectives and Recommendations

Future studies should include experimental validation of the identified pathways, particularly those linked to ECM remodeling and neurotransmitter signaling, using functional assays to dissect their roles in H-TNBC biology. In addition, future work should validate immune deconvolution findings through complementary experimental methods such as flow cytometry or single-cell RNA-seq, which would allow a more precise characterization of immune cell dynamics within the tumor microenvironment. Moreover, immunophenotyping and spatial transcriptomics could further clarify the cellular interactions driving the immune landscape observed in hereditary tumors. Clinically, incorporating germline and somatic profiles into therapeutic decision-making could refine patient stratification and guide personalized immunotherapy approaches. Lastly, expanding analyses to larger and multi-ancestry cohorts will help confirm the influence of genetic background on the tumor immune microenvironment and treatment outcomes. Finally, future validation efforts should integrate datasets beyond TCGA, including cohorts with comprehensive germline variant annotation covering a broader spectrum of hereditary breast cancer genes. This will allow more accurate cross-population comparisons and enhance the representativeness of hereditary TNBC beyond *BRCA1/2* mutation carriers. In addition, future studies employing breast cancer cell lines or patient-derived models could help bridge the transcriptomic findings with functional phenotypes, providing mechanistic insights into how gene expression alterations shape tumor behavior and immune modulation.

### 5.2. Limitations

We acknowledge a limitation in sample size, which may limit the generalizability of the findings and reduce the statistical power to detect subtle associations or interactions. In addition, the definition of hereditary tumors in this study was based on the presence of P/LP variants in a subset of genes associated with different DNA repair pathways (HRR, BER, and MMR), which differs from other studies that considered only *BRCA1/2*. This broader categorization may partly explain the molecular and immunological differences observed, but it also complicates direct comparisons with external datasets, such as TCGA, where hereditary cases were restricted to *BRCA1/2* mutation carriers. While the use of high-dimensional RNA-seq data and robust bioinformatic methods strengthens the reliability of our findings, larger cohorts with harmonized genetic definitions are needed to validate these results. Moreover, the cross-sectional nature of the study precludes the assessment of longitudinal clinical outcomes, such as therapy response or survival, which remain to be evaluated in prospective studies integrating transcriptomic and clinical data. Finally, single-cell or spatial transcriptomic approaches could provide finer resolution of tumor–immune interactions in future work. Additionally, the pathways and gene signatures identified through RNA-seq warrant further experimental validation such as quantitative PCR, immunohistochemistry, or functional assays to confirm and extend the bioinformatic findings presented here. Furthermore, immune profiling in this study was based on transcriptomic deconvolution using XCell and CIBERSORTx, which, while robust for bulk RNA-seq data, cannot fully capture the heterogeneity or spatial context of immune cell populations. Direct experimental approaches—such as flow cytometry or single-cell RNA-seq—are needed to validate and expand these observations in future work.

## Figures and Tables

**Table 1:** Clinical and pathological characteristics of hereditary and sporadic TNBC cases in the Colombian cohort.

Characteristic	Hereditary	Sporadic	*p* Value
	N = 20 (%)	N = 42 (%)	
**Age**			0.334 *^1^*
≤50	9 (45%)	23 (62%)	
>50	11 (55%)	14 (38%)	
**BMI**			0.670 *^2^*
Normal	10 (50%)	16 (43%)	
Obesity	2 (10%)	7 (19%)	
Overweight	8 (40%)	14 (38%)	
**Menopausal state**			0.334 *^1^*
Postmenopausal	11 (55%)	14 (38%)	
Premenopausal	9 (45%)	23 (62%)	
**Tumor Differentiation**			0.357 *^2^*
Poorly Differentiated	17 (89%)	27 (75%)	
Moderately Differentiated	2 (11%)	9 (25%)	
**Ki67**			0.536 *^1^*
>50%	14 (74%)	28 (85%)	
≤50%	5 (26%)	5 (15%)	
**Laterality**			0.617 *^1^*
Right	6 (30%)	15 (41%)	
Left	14 (70%)	22 (59%)	
**T**			0.524 *^1^*
Tumor ≤ 5 cm	12 (60%)	17 (47%)	
Tumor > 5 cm	8 (40%)	19 (53%)	
**N**			0.028 *^2^*
Lymph node involvement (N1-N2)	11 (55%)	30 (86%)	
No lymph node metastasis	9 (45%)	5 (14%)	
**M**			0.992 *^2^*
Metastasis	3 (15%)	4 (11%)	
No Metastasis	17 (85%)	31 (89%)	
**Metastasis location**			0.050 *^2^*
Regional lymph nodes	0 (0%)	4 (100%)	
Pleura	1 (33%)	0 (0%)	
Lung	2 (67%)	0 (0%)	
**AJCC Stage**			0.126 *^2^*
Early	12 (60%)	12 (35%)	
Advanced	5 (25%)	18 (53%)	
Metastasis	3 (15%)	4 (12%)	

*^1^ Chi-squared; ^2^ Fisher’s exact test. p-values ≥ 0.05 were considered not significant. BMI = Body Mass Index. T = Tumor size. N = Lymph node involvement. M = Metastasis. AJCC = American Joint Committee on Cancer.*

**Table 2:** Genes selected by LASSO logistic regression distinguishing hereditary from sporadic TNBC.

Category	Functional Group	Enriched Terms
GO: Biological Process (BP)	Immune response	Regulation of IL17 production Negative regulation of response to cytokine stimulus Myeloid leukocyte cytokine production
	Cellular processes	Positive regulation of cell–cell adhesion Regulation of protein secretion Cell activation involved in immune response
GO: Molecular Function (MF)	Receptor binding	Cytokine receptor binding, receptor ligand activity
	Enzymatic activity	Transferase activity, kinase activity
KEGG Pathway	KEGG pathway	Cytokine–cytokine receptor interaction, TNF signaling pathway, NF-kappa B signaling pathway, pathways in cancer, apoptosis

## Data Availability

The data supporting the findings of this study are available from the corresponding author upon reasonable request. Due to patient privacy and ethical restrictions, raw sequencing data cannot be made publicly available.

## Supplementary Materials

The following supporting information can be downloaded at https://www.mdpi.com/article/10.3390/biology14121706/s1. Figure S1: Quality Control of RNA-Seq Data.; Figure S2: Distribution of pathogenic and likely pathogenic (P/LP) variants in 20 patients with hereditary TNBC. Figure S3: Functional Enrichment Analysis in Colombian women cohort — Downregulated genes. Table S1: TruSight™ Hereditary Cancer Panel; Table S2: Comparison of Immunophenoscore (IPS) between hereditary and sporadic TNBC in Colombian and TCGA cohorts. Table S3: Genes selected by LASSO logistic regression distinguishing hereditary from sporadic TNBC.

## Abbreviations

The following abbreviations are used in this manuscript:

BC	Breast Cancer
TNBC	Triple-Negative Breast Cancer
H-TNBC	Hereditary Triple-Negative Breast Cancer
S-TNBC	Sporadic Triple-Negative Breast Cancer
ER	Estrogen Receptor
PR	Progesterone Receptor
HER2	Human Epidermal Growth Factor Receptor 2
HRR	Homologous Recombination Repair
TILs	Tumor-Infiltrating Lymphocytes
TME	Tumor Microenvironment
Tcm	Central Memory CD4+ T cells
NCCN	National Comprehensive Cancer Network
P	Pathogenic Variants
LP	Likely Pathogenic Variants
FFPE	Formalin-Fixed, Paraffin-Embedded
RSEM	RNA-Seq by Expectation-Maximization
TMM	Trimmed Mean of M-values
CPM	Counts Per Million
DEGs	Differentially Expressed Genes
GO	Gene Ontology
KEGG	Kyoto Encyclopedia of Genes and Genomes
TPM	Transcripts Per Million
TCGA	The Cancer Genome Atlas
NCI-Col	Colombian National Cancer Institute
BP	Biological Process
CC	Cellular Component
MF	Molecular Functions
ECM	Extracellular Matrix
TMB	Tumor Mutational Burden
Tregs	Regulatory T cells
GABA	Gamma-aminobutyric acid
