# Supplementary material for: Comparative Genomic and Microenvironmental Profiles of Hereditary and Sporadic TNBC in Colombian Women
Source: Biology (Basel). 2025 Nov 30;14(12):1706. doi: 10.3390/biology14121706 (PMC12730083; doi:10.3390/biology14121706)
Supplement: Supplementary file 1 [file biology-14-01706-s001.zip › biology-3918040-supplementary.pdf]

Research Article | Supplementary materials

Supplementary Table S1: TruSight™ Hereditary Cancer Panel

Gen	Chromosome
AIP	11
ALK	2
APC	5
ATM	11
BAP1	3
BARD1	2
BLM	15
BMPR1A	10
BRCA1	17
BRCA2	13
BRIP1	17
BUB1B	15
CASR	3
CDC73	1
CDH1	16
CDK4	12
CDKN1B	12
CDKN1C	11
CDKN2A	9
CEBPA	19
CEP57	11
CHEK2	22
CYLD	16
DDB2	11
DICER1	14
DIS3L2	2
EGFR	7
EPCAM	2
ERCC2	19
ERCC3	2
ERCC4	16
ERCC5	13
EXT1	8

Gen	Chromosome
FANCB	X
FANCC	9
FANCD2	3
FANCE	6
FANCF	11
FANCG	9
FANCI	15
FANCL	2
FANCM	14
FH	1
FLCN	17
GATA2	3
GNAS	20
GPC3	X
HNF1A	12
HRAS	11
KIT	4
MAX	14
MEN1	11
MET	7
MLH1	3
MRE11A	11
MSH2	2
MSH6	2
MUTYH	1
NBN	8
NF1	17
NF2	22
NSD1	5
PALB2	16
PDE4D	5
PHOX2B	4
PMS1	2

Gen	Chromosome
PPM1D	17
PRF1	10
PRKAR1A	17
PTCH1	9
PTEN	10
RAD50	5
RAD51C	17
RAD51D	17
RB1	13
RECQL4	8
RET	10
RHBDF2	17
RUNX1	21
SBDS	7
SDHA	5
SDHAF2	11
SDHB	1
SDHC	1
SDHD	11
SLX4	16
SMAD4	18
SMARCB1	22
STK11	19
SUFU	10
TMEM127	2
TP53	17
TSC1	9
TSC2	16
VHL	3
WRN	8
WT1	11
XPA	9
XPC	3

EXT2	11
EZH2	7
FANCA	16

PMS2	7
POLD1	19
POLE	12

Supplementary Figure S1: Quality Control of RNA-Seq Data

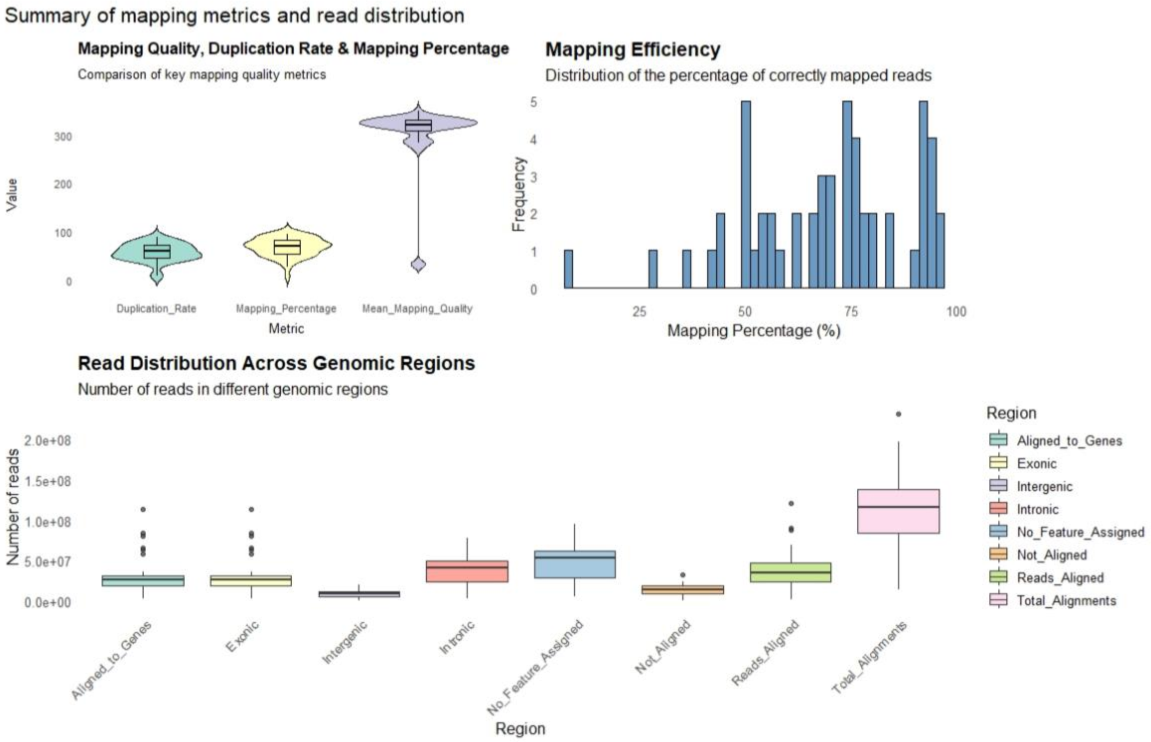

Figure S1: Summary of RNA-Seq mapping quality metrics and read distribution across genomic regions in the Colombian TNBC cohort. (A) Violin plots showing the distribution of mapping quality, duplication rate, and overall mapping percentage across samples. (B) Histogram representing the frequency distribution of correctly mapped reads (mapping efficiency). (C) Boxplots illustrating the number of reads assigned to different genomic regions, including exonic, intronic, and intergenic fractions. Quality metrics were obtained from Qualimap RNA-Seq reports and summarized in R.

Supplementary Figure S2: Distribution of pathogenic and likely pathogenic (P/LP) variants in 20 patients with hereditary TNBC.

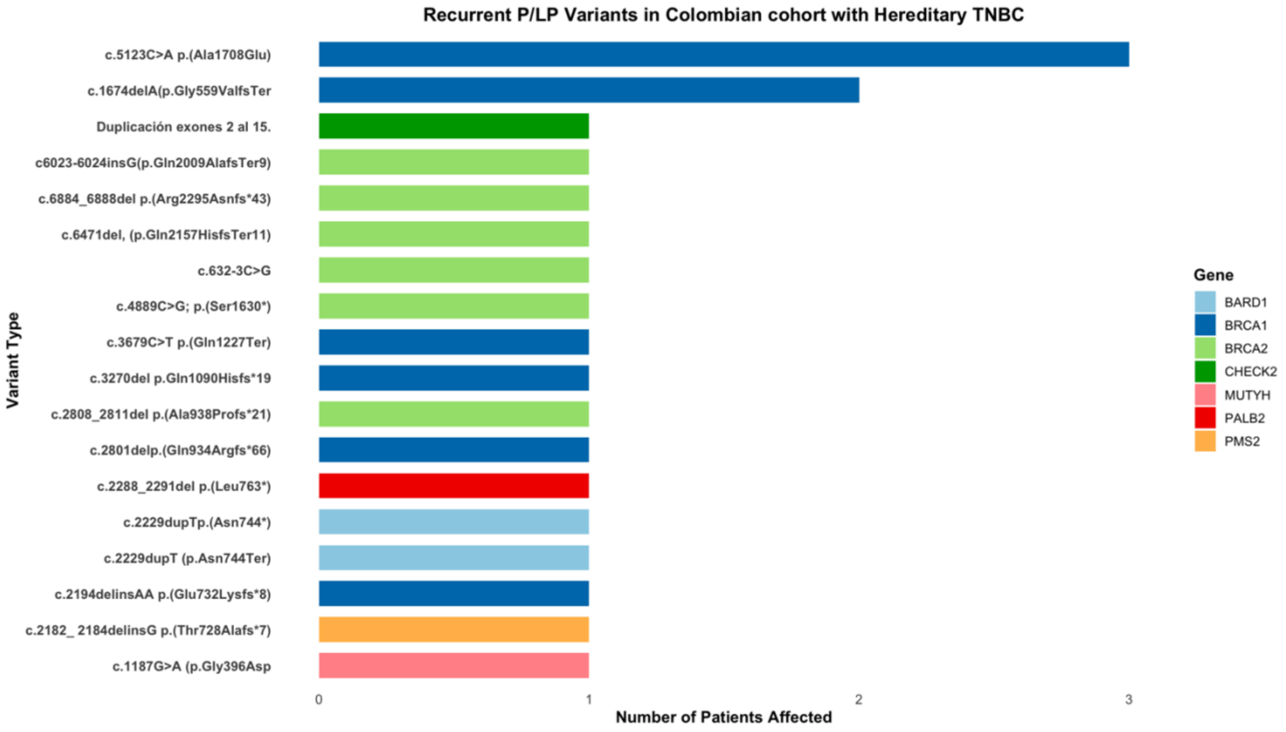

Figure S2: A total of 13 distinct P/PL variants were identified. The most frequently mutated gene was *BRCA1* (dark blue bars) present in 10 patients with 6 unique variants. The second most frequently affected gene was *BRCA2* (light green bars), present in 6 patients. *BARD1* (light blue bars) was mutated in 2 patients. All other variants were identified in a single patient each.

Supplementary Figure S3: Functional Enrichment Analysis in Colombian women cohort – Downregulated genes.

2A: GO: Biological Process – Downregulated DEGs

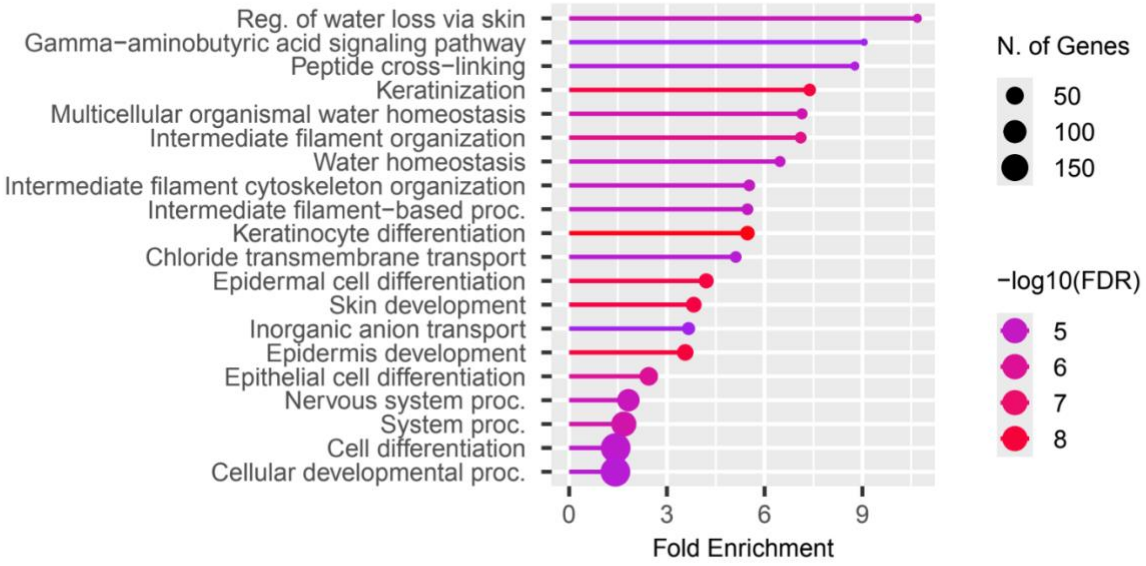

2B: GO: Cellular Components – Downregulated DEGs

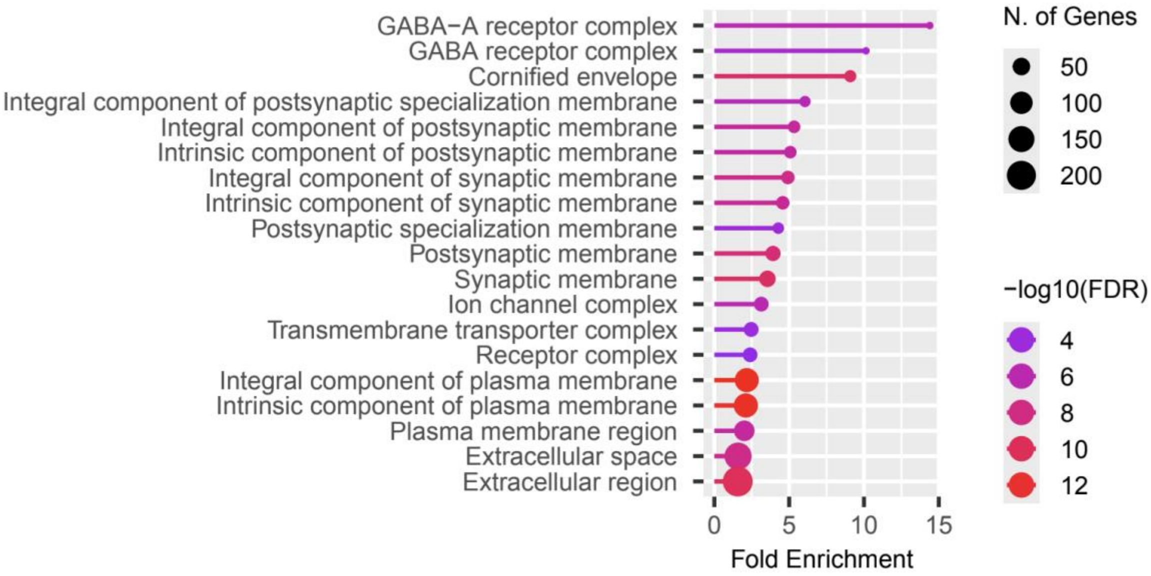

2C: GO: Molecular Functions – Downregulated DEGs

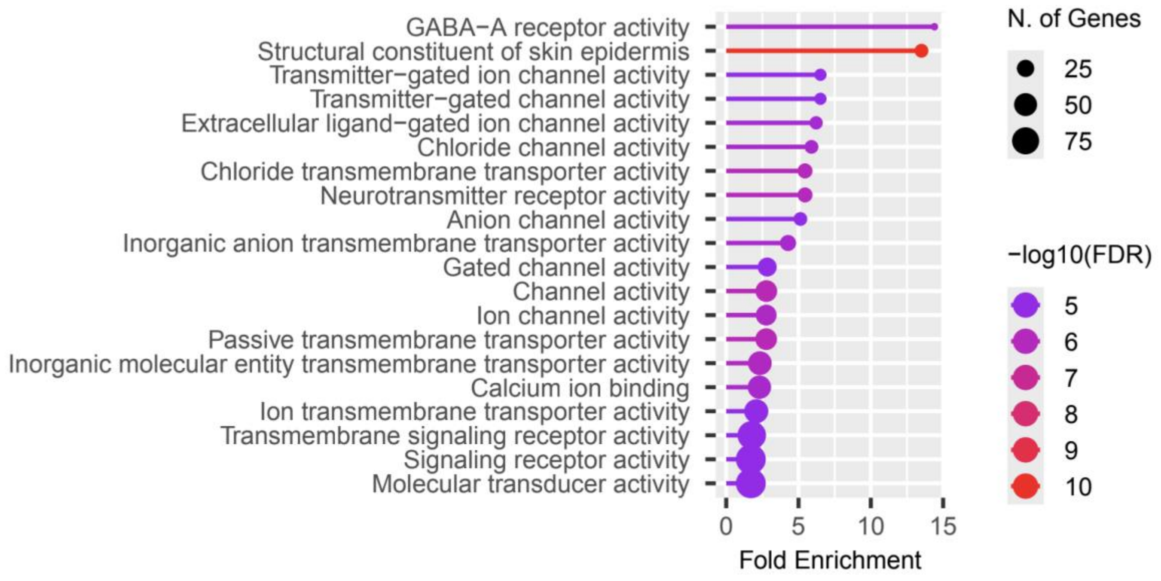

2D: KEGG Pathways – Downregulated DEGs

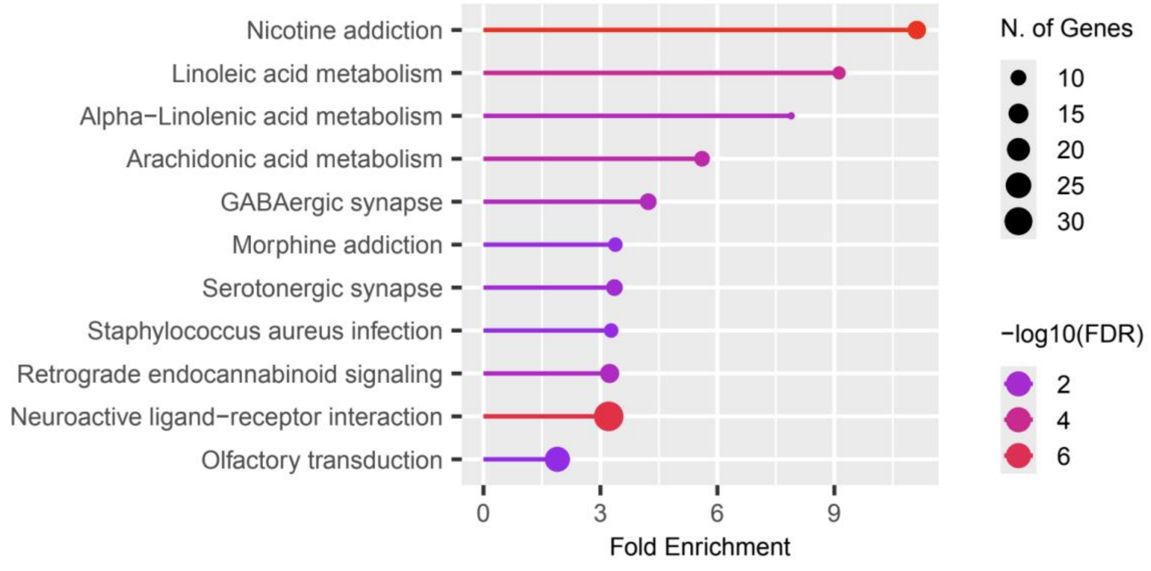

Figure S3. Functional enrichment compared H-TNBC vs S-TNBC. (2A) GO Biological Process. (2B) GO Cellular Component. (2C) Molecular Functions and (2D) KEGG Pathways. ach dot represents an enriched category, with size indicating the number of genes and color representing statistical significance.

Supplementary Table S2: Comparison of Immunophenoscore (IPS) between hereditary and sporadic TNBC in Colombian and TCGA cohorts.

IPS	TCGA Cohort			Colombian Cohort		
	**H-TNBC**	**S-TNBC**	***p value***	**H-TNBC**	**S-TNBC**	***p value***
**High IPS (%)**	3 (37.5%)	4 (50 %)	*1**	6 (30%)	7 (16.7%)	*0.32**
**Low IPS (%)**	5 (62.5%)	4 (50 %)		14 (70%)	35 (83.3%)	

**Fisher exact test*

Supplementary Table S3: Genes selected by LASSO logistic regression distinguishing hereditary from sporadic TNBC.

Gene	Coefficient
GSTA1	0,421
FOXQ1	0,352
NPTX2	0,156
IL6	0,128
FOXC2	0,083
MMP13	0,045
MSLN	0,013
HLF	-0,258
AMPD1	-0,227
EPHA6	-0,221
CYP2E1	-0,188
B4GALNT2	-0,183
AMH	-0,158
DSG1	-0,138
PLCH2	-0,130
ENDOU	-0,114
IL36RN	-0,099
EEF1A2	-0,089
CHIT1	-0,066
DDX25	-0,059
KRT9	-0,054
TPSD1	-0,029
BTN1A1	-0,028
